# Cluster identification, selection, and description in cluster randomized crossover trials: the PREP-IT trials

**DOI:** 10.1186/s13063-020-04611-9

**Published:** 2020-08-12

**Authors:** Sheila Sprague, Taryn Scott, Shannon Dodds, David Pogorzelski, Paula McKay, Anthony D. Harris, Amber Wood, Lehana Thabane, Mohit Bhandari, Samir Mehta, Greg Gaski, Christina Boulton, Francesc Marcano-Fernández, Ernesto Guerra-Farfán, Joan Hebden, Lyndsay M. O’Hara, Gerard P. Slobogean, on behalf of the PREP-IT Investigators

**Affiliations:** 1 Department of Surgery, Division of Orthopaedic Surgery, McMaster University, 293 Wellington Street North, Suite 110, Hamilton, ON L8L 8E7 Canada; 2 Department of Health Research Methods, Evidence, and Impact, McMaster University, 293 Wellington St. N., Suite 110, Hamilton, ON L8L 8E7 Canada; 3 Department of Epidemiology and Public Health, University of Maryland School of Medicine, Baltimore, MD USA; 4 Association of periOperative Registered Nurses, Denver, CO USA; 5 Department of Orthopaedic Surgery, University of Pennsylvania Perelman School of Medicine, Philadelphia, PA USA; 6 Department of Orthopaedic Surgery, Indiana University School of Medicine, Indianapolis, IN USA; 7 Department of Orthopaedics, Banner Health and the University of Arizona-Tucson, Tucson, AZ USA; 8 Orthopedic Department, Parc Taulí Hospital Universitari, Institut d’Investigació i Innovació Parc Taulí I3PT, Universitat Autònoma de Barcelona, Sabadell, Spain; 9 Department of Orthopaedic Surgery and Traumatology, University Hospital Vall d’Hebron, Barcelona, Spain; 10 Department of Medicine, University of Maryland School of Medicine, Baltimore, MD USA; 11 Department of Orthopaedics, University of Maryland, R Adams Cowley Shock Trauma Center, Baltimore, MD USA

**Keywords:** Cluster, Randomized crossover, Pragmatic, Cluster characteristics, Orthopedic, Surgical site infection

## Abstract

**Background:**

In cluster randomized crossover (CRXO) trials, groups of participants (i.e., clusters) are randomly allocated to receive a sequence of interventions over time (i.e., cluster periods). CRXO trials are becoming more comment when they are feasible, as they require fewer clusters than parallel group cluster randomized trials. However, CRXO trials have not been frequently used in orthopedic fracture trials and represent a novel methodological application within the field. To disseminate the early knowledge gained from our experience initiating two cluster randomized crossover trials, we describe our process for the identification and selection of the orthopedic practices (i.e., clusters) participating in the PREP-IT program and present data to describe their key characteristics.

**Methods:**

The PREP-IT program comprises two ongoing pragmatic cluster randomized crossover trials (Aqueous-PREP and PREPARE) which compare the effect of iodophor versus chlorhexidine solutions on surgical site infection and unplanned fracture-related reoperations in patients undergoing operative fracture management. We describe the process we used to identify and select orthopedic practices (clusters) for the PREP-IT trials, along with their characteristics.

**Results:**

We identified 58 potential orthopedic practices for inclusion in the PREP-IT trials. After screening each practice for eligibility, we selected 30 practices for participation and randomized each to a sequence of interventions (15 for Aqueous-PREP and 20 for PREPARE). The majority of orthopedic practices included in the Aqueous-PREP and PREPARE trials were situated in level I trauma centers (100% and 87%, respectively). Orthopedic practices in the Aqueous-PREP trial operatively treated a median of 149 open fracture patients per year, included a median of 11 orthopedic surgeons, and had access to a median of 5 infection preventionists. Orthopedic practices in the PREPARE trial treated a median of 142 open fracture and 1090 closed fracture patients per year, included a median of 7.5 orthopedic surgeons, and had access to a median of 6 infection preventionists.

**Conclusions:**

The PREP-IT trials provide an example of how to follow the reporting standards for cluster randomized crossover trials by providing a clear definition of the cluster unit, a thorough description of the cluster identification and selection process, and sufficient description of key cluster characteristics.

**Trial registration:**

Both trials are registered at ClinicalTrials.gov (A-PREP: NCT03385304 December 28, 2017, and PREPARE: NCT03523962 May 14, 2018).

## Background

In clinical research, large high-quality randomized controlled trials (RCTs) are considered the highest level of evidence to determine the effectiveness of an intervention [1]. While most RCTs follow a parallel group design and randomize individual participants to one or more intervention and control groups, there are certain situations in which randomization at this level is not feasible or practical [2]. Cluster randomized trials overcome these challenges by randomizing predetermined groups of participants (i.e., clusters) to interventions [3]. In cluster randomized crossover (CRXO) trials, each cluster serves as their own control group by participating in both the treatment and control arm at least once at various periods of time throughout the trial. An advantage to this design [CRXO] over the parallel group cluster randomized trial is that fewer clusters are required to achieve statistical power.

Researchers undertaking CRXO trials must ensure that they take into consideration multiple differences in the conduct and reporting between RCTs and CRXO trials [4, 5]. One of these fundamental differences is the requirement to provide information on the characteristics and flow of clusters throughout the conduct of a CRXO trial (i.e., enrolment, allocation, follow-up, analysis), as described in the CONSORT extension for cluster randomized trials [4]. This is accomplished by including a priori cluster selection criteria (similar to participant eligibility criteria) and a cluster flow diagram that shows the cluster selection and flow over the duration of the trial (similar to the patient flow diagram). This information enables knowledge users to assess the generalizability of the trial and determine if the results are applicable to their setting and practice.

To disseminate the early knowledge gained from our experience initiating two large infection prevention CRXO trials in orthopedic fracture patients, we describe our cluster identification and selection process. Additionally, we present data on the key characteristics of the clusters to illustrate the level of data collection that is required for appropriate reporting in CRXO trials.

## Methods

### The PREP-IT program

**Fig. 1:**
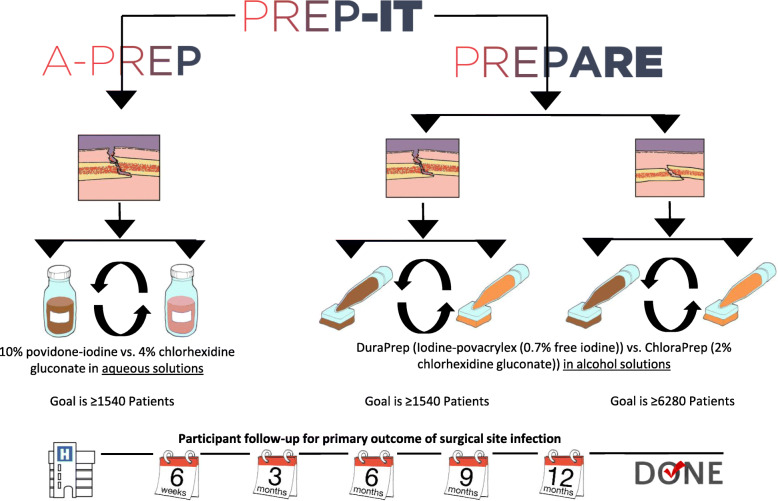
The PREP-IT research program

The Program of Randomized trials to Evaluate Pre-operative antiseptic skin solutions in orthopaedic Trauma (PREP-IT) aims to determine the effectiveness of iodophor compared to chlorhexidine solutions at reducing surgical site infection (SSI) and unplanned fracture-related reoperations in fracture surgery patients. The PREP-IT program includes two ongoing multi-center pragmatic CRXO trials that study the effects of four antiseptic solutions in three independent populations of surgically treated fracture patients: the Aqueous-PREP (A Pragmatic Randomized trial Evaluating Pre-operative aqueous antiseptic skin solutions in open fractures) trial and the PREPARE (A Pragmatic Randomized trial Evaluating Pre-operative Alcohol skin solutions in Fractured Extremities) trial (Fig. 1). The Aqueous-PREP trial compares 4% aqueous chlorhexidine versus 10% povidone-iodine in open extremity fracture patients. The PREPARE trial compares 2% chlorhexidine in 70% isopropyl alcohol (ChloraPrep™) versus 0.7% iodine povacrylex in 74% isopropyl alcohol (DuraPrep™) in both open extremity fracture patients and patients with closed lower extremity or pelvic fractures. Both trials follow a single master protocol which has been described in a previous manuscript [6].

Briefly, the order of treatment allocation for each orthopedic practice (i.e., cluster) was randomly assigned using a computer-generated randomization table. Simple, non-stratified randomization was used. Cluster randomization is performed centrally at the Methods Centre by personnel who are unaware of the cluster characteristics. Randomization is performed when a cluster has received local ethics approval and their clinical trial agreement is in place.

Each orthopedic practice starts with the surgical preparation solution to which they were initially allocated and subsequently crosses over to the other solution for their second cluster period. This process of alternating treatments repeats approximately every 2 months as dictated by the initial randomization. This process of alternating treatment periods (crossovers) will continue until the minimum sample size is achieved for each fracture population and the study’s planned recruitment period is completed. Clinical sites must complete at least two treatment periods (one crossover), and the majority of clinical sites will complete 12 treatment periods (11 crossovers), and 24 months of enrollment.

For both trials, the primary outcome is SSI as defined by the Centers for Disease Control and Prevention (CDC) [7] which includes superficial incisional SSI within 30 days and deep incisional or organ/space SSI within 90 days of definitive fracture management surgery. We continue to follow participants for SSI for 12 months to inform our sensitivity analyses. The secondary outcome is unplanned fracture-related reoperations within 12 months to manage infection, wound healing problems, and fracture healing problems. The Aqueous-PREP trial will enroll a minimum of 1540 open fracture patients, and PREPARE will enroll a minimum of 1540 open fracture patients and 6280 closed fracture patients.

### Cluster definition

We defined clusters as orthopedic practices within participating hospitals. Each participating hospital has only one participating orthopedic practice. Clustering occurs at the level of the orthopedic practice as only orthopedic surgeons were required to follow the study protocol and use the randomized surgical preparation solution. During the design phase, we considered defining clusters as either the hospitals with which orthopedic practices were affiliated or the operating rooms used by orthopedic practices; however, we decided against these definitions as other specialities associated with the hospital or operating rooms were not required to follow the study protocol.

### Cluster eligibility criteria

To be eligible for inclusion in the PREP-IT trials, orthopedic practices were required to meet the predetermined eligibility criteria as specified in the trial protocols. Specifically, the inclusion criteria were as follows: (1) adequate research personnel infrastructure to manage the study, (2) adequate open fracture volume and closed lower extremity and pelvic fracture volume to complete enrollment within the study timeline (i.e., a minimum of 77 open fractures (for Aqueous-PREP and PREPARE) and 314 closed lower extremity fractures per year (for PREPARE only)), (3) commitment from all or most orthopedic surgeons to participate in the trial, and (4) ability to use the two skin preparation solutions. The exclusion criteria were as follows: (1) lack of interest in the trial; (2) anticipated challenges with complying with the protocol; (3) conflicting studies, in the judgment of the Principal Investigators, that would inhibit patient participation; (4) budgeting or contract constraints; and (5) conflicting roles within the trials (e.g., member of the Data and Safety Monitoring Board).

### Cluster identification and selection

We identified orthopedic practices through professional contacts with the PREP-IT Principal Investigators. Each orthopedic practice received an email containing information about the PREP-IT trials, as well as an invitation to apply to participate in either one or both trials. For orthopedic practices who declined this invitation, we identified and documented the reason for exclusion using our predetermined eligibility criteria. Orthopedic practices who expressed a desire to participate were required to complete a feasibility questionnaire. This questionnaire consisted of 17 questions and assessed the orthopedic practice’s research experience and infrastructure, fracture volume, current practice patterns, and interest in participating in one or both trials. We used the responses to the feasibility questionnaire to preliminarily screen orthopedic practices for eligibility. Orthopedic practices that were found to be ineligible through this preliminary screen were notified, and the reason for exclusion was documented. Orthopedic practices that were eligible to participate following the preliminary screen were invited to participate in a series of teleconferences with the PREP-IT Principal Investigators and study personnel. The purpose of these calls was to review trial and clinical practice logistics in detail and to confirm whether each orthopedic practice met the eligibility criteria for participation. For any orthopedic practice that was determined to be ineligible through these calls, we documented the reason for exclusion. Orthopedic practices that met all eligibility criteria were selected to participate.

### Collection of cluster characteristics

Upon selection, orthopedic practices were required to complete a cluster definition questionnaire. This questionnaire, which is comprised of 44 questions, captures important data on hospital characteristics, current surgeon preferences and practices for pre-operative surgical preparation techniques, and infection co-interventions known to reduce the incidence of SSIs. Each orthopedic practice’s questionnaire data is updated every 4 months over the enrollment and follow-up periods.

### Statistical analyses

Our statistical analysis plan was determined a priori. We included data from the orthopedic practices currently enrolling participants into the Aqueous-PREP trial and the PREPARE trial. We used descriptive statistics to summarize all characteristics of the orthopedic practices (frequencies and percentages for categorical variables, and medians and interquartile ranges (IQRs) for continuous variables). We used Microsoft Excel 2016 to conduct all statistical analyses.

## Results

### Cluster selection

**Fig. 2:**
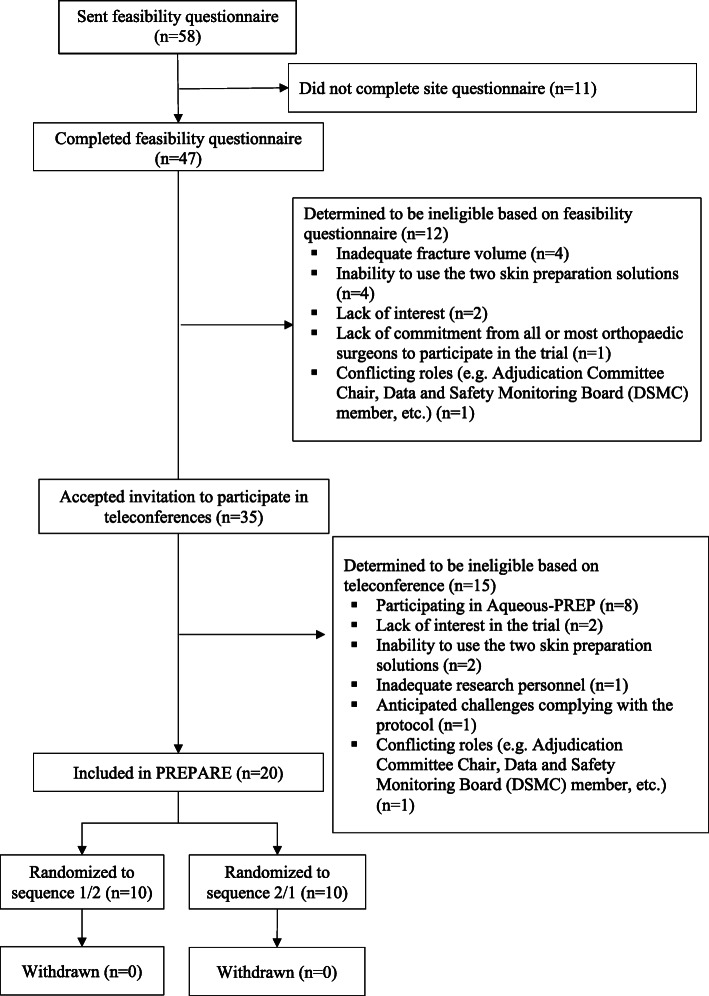
PREPARE CONSORT flowchart for clusters

**Fig. 3:**
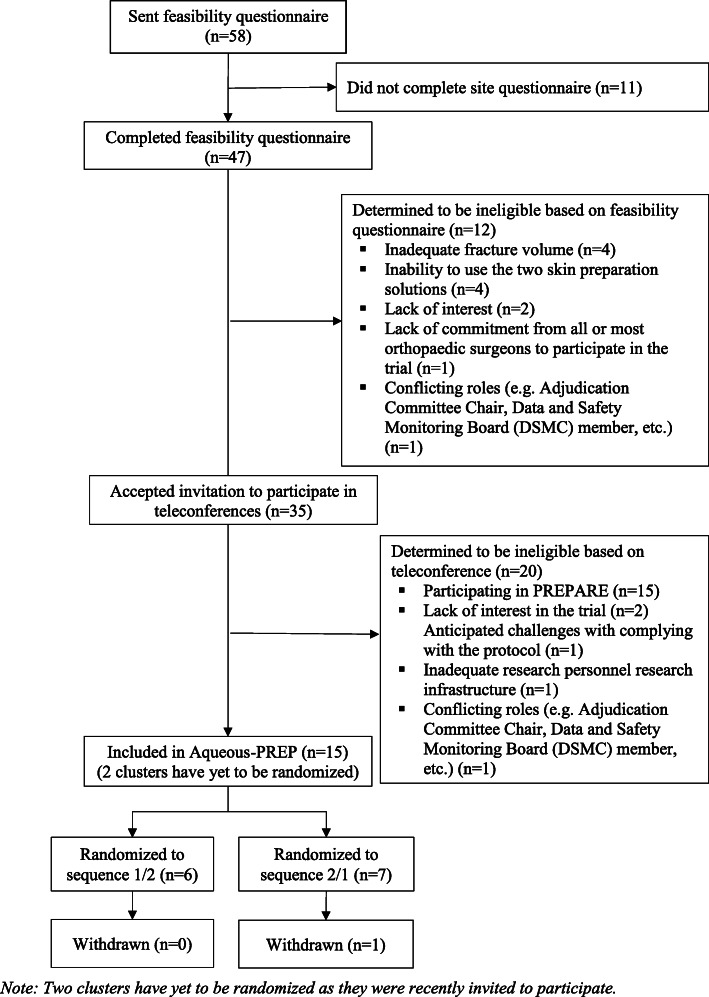
Aqueous-PREP CONSORT flowchart for clusters

Of the 58 potentially eligible orthopedic practices that we identified, 47 (81%) accepted our invitation to complete a feasibility questionnaire and 11 (19%) declined our invitation and were excluded due to a lack of interest in the trial (Figs. 2 and 3). Of the 47 practices that completed the feasibility questionnaire, 12 were determined to be ineligible and 35 were invited to participate in a teleconference with the Principal Investigators and study team to further assess eligibility. Of the 35 orthopedic practices that participated in the teleconferences, 5 were determined to be ineligible, 5 were selected to participate in both the Aqueous-PREP and PREPARE trials, 10 in the Aqueous-PREP trial only, and 15 in the PREPARE trial only. Specific reasons for orthopedic practice exclusions for each trial are documented in Figs. 2 and 3. One of the practices participating in the Aqueous-PREP trial was discontinued post-randomization due to an inability to follow the trial protocol. Specifically, this cluster did not meet the a priori threshold of treatment compliance (e.g., 90% of patients receive the allocated antiseptic solution) and the clinical site was unable to complete the case report forms, which led to incomplete data submission. The clinical site enrolled 14 participants and was withdrawn within 2 months of initiation. Therefore, there are 14 orthopedic practices in the Aqueous-PREP trial and 20 orthopedic practices in the PREPARE trial.

### Characteristics of participating clusters

Table 1 details the name and location of the hospital associated with each orthopedic practice participating in the Aqueous-PREP and PREPARE trials, as well as the intervention sequence to which they were randomized. The majority of orthopedic practices are affiliated with hospitals that are level I trauma centers and owned by a non-profit, non-religious organization (Table 2). The median population served is 1,625,000 for orthopedic practices participating in the Aqueous-PREP trial and 2,400,000 for the PREPARE trial. The characteristics of the withdrawn cluster are presented in the Appendix.

**Table 1:** Clusters and location

Hospital (cluster) name	Location	Randomization sequence (1/2 vs. 2/1)	Trial
University of Maryland School of Medicine, R Adams Cowley Shock Trauma Center	Baltimore, MD	2/1 (Aqueous-PREP); 1/2 (PREPARE)	Aqueous-PREP and PREPARE
Greenville Health System	Greenville, SC	2/1 (Aqueous-PREP); 2/1 (PREPARE)	Aqueous-PREP and PREPARE
Hamilton Health Sciences – General Site	Hamilton, ON	1/2 (Aqueous-PREP); 2/1 (PREPARE)	Aqueous-PREP and PREPARE
IU Health Methodist Hospital	Indianapolis, IN	1/2 (Aqueous-PREP); 1/2 (PREPARE)	Aqueous-PREP and PREPARE
San Antonio Military Medical Center	San Antonio, TX	1/2 Aqueous-PREP; 1/2 (PREPARE)	Aqueous-PREP and PREPARE
Banner – University Medical Center Tucson	Tucson, AZ	1/2	Aqueous-PREP
Hospital Universitari Parc Tauli	Barcelona, Spain	To be randomized	Aqueous-PREP
McGovern Medical School at UTHealth Houston	Houston, TX	1/2	Aqueous-PREP
The CORE Institute	Phoenix, AZ	2/1	Aqueous-PREP
University of California, San Francisco	San Francisco, CA	1/2	Aqueous-PREP
University of Florida	Gainesville, FL	2/1	Aqueous-PREP
Vall d’Hebron Hospital	Barcelona, Spain	To be randomized	Aqueous-PREP
Vanderbilt Medical Center	Nashville, TN	2/1	Aqueous-PREP
Wright State University	Dayton, OH	2/1	Aqueous-PREP
Brigham and Women’s Hospital	Boston, MA	1/2	PREPARE
Carolina’s Medical Center	Charlotte, NC	2/1	PREPARE
Dartmouth-Hitchcock Medical Center	Lebanon, NH	2/1	PREPARE
Duke University Hospital	Durham, NC	2/1	PREPARE
Inova Fairfax Medical Campus	Fairfax, VA	2/1	PREPARE
Massachusetts General Hospital	Boston, MA	1/2	PREPARE
MetroHealth Medical Center	Cleveland, OH	1/2	PREPARE
Royal Columbian Hospital	New Westminster, BC	2/1	PREPARE
Regional Medical Center of San Jose	San Jose, CA	1/2	PREPARE
Sanford Health	Sioux Falls, SD	1/2	PREPARE
University of Maryland Capital Regional Health	Cheverly, MD	2/1	PREPARE
University of Mississippi Medical Center	Jackson, MS	2/1	PREPARE
University of Pennsylvania	Philadelphia, PA	1/2	PREPARE
University of Utah	Salt Lake City, UT	2/1	PREPARE
Wake Forest Baptist Hospital	Winston-Salem, NC	1/2	PREPARE

**Table 2:** Cluster characteristics

Characteristic	Aqueous-PREP, ***N*** = 14*	PREPARE, ***N*** = 20
Level of trauma care provided, *n* (%)		
Level I	14 (100)	17 (85)
Level II	0 (0)	3 (15)
Ownership of hospital, *n* (%)		
Non-profit, not religious order affiliated	8 (57)	15 (75)
Government	4 (29)	4 (20)
Private	1 (7)	1 (5)
Private administration with public funding	1 (7)	
Hospital affiliation, *n* (%)		
Independent, free-standing	2 (14)	1 (5)
Multi-facility organization (chain)	5 (36)	5 (25)
Hospital system, attached	4 (29)	7 (35)
Hospital system, free-standing	3 (21)	7 (35)
Population size served, median (IQR)	1,625,000 (3,735,699)	2,400,000 (2,875,319)
Number of inpatient beds, median (IQR)	679 (287.3)	616 (388.5)
Number of operating rooms, median (IQR)	21 (18)	31 (22.5)
Number of beds in intensive care unit, median (IQR)	48.5 (44.8)	74 (81.5)
Hospital is a primary teaching hospital for a medical school, *n* (%)	14 (100)	16 (80)

*IQR* interquartile range

*The cluster that was withdrawn is not included

Orthopedic practices participating in the Aqueous-PREP trial treat a median of 149 open fractures annually and include a median of 11 orthopedic surgeons who treat fractures and 8.5 who take trauma calls (Table 3). Orthopedic practices participating in the PREPARE trial treat a median of 142 open fractures annually and 1090 closed fractures annually. These orthopedic practices include a median of 7.5 orthopedic surgeons who treat fracture patients and 9.5 who take trauma calls.

**Table 3:** Orthopedic characteristics

Characteristic	Aqueous-PREP, ***N*** = 14*	PREPARE, ***N*** = 20
Number of orthopedic surgeons who treat fracture patients, median (IQR)	11 (11.3)	7.5 (7)
Number of orthopedic surgeons who take trauma calls, median (IQR)	8.5 (13)	9.5 (9.5)
Orthopedic training programs, *n* (%)**		
Orthopedic fellowship	13 (93)	17 (85)
Orthopedic residency	14 (100)	18 (90)
Orthopedic clerkship	14 (100)	17 (85)
None of above	0 (0)	1 (5)
Annual number of operatively managed open fractures, median (IQR)	149 (125)	142 (185)
Annual number of operatively managed closed fractures, median (IQR)	1200 (828.8)	1090 (630.5)
Annual number of operatively managed closed lower extremity and pelvic fractures, median (IQR)	–	725 (435)
Number of operating rooms used to treat fractures, median (IQR)	4 (2)	4 (5.5)

*IQR* interquartile range

*The cluster that was withdrawn is not included

**Percentages do not sum to 100 as categories are not mutually exclusive

Participating orthopedic practices employ a variety of surgical infection prevention measures (Table 4). Specifically, practices participating in the Aqueous-PREP trial have a median of 2.2 infection preventionists per 250 hospital beds. Sixty-four percent of hospitals (*n* = 9) perform active surveillance for methicillin-resistant *Staphylococcus aureus* (MRSA) in open fracture patients. None of the clinical sites perform active surveillance for vancomycin-resistant *Enterococcus* (VRE) in open fracture patients. Sixty-four percent of practices (*n* = 9) have a decolonization protocol for MRSA. All practices have some infection control parameters in place in their operating rooms (e.g., high-efficiency particulate air (HEPA) filters, positive pressure operating rooms, air changes per hour, temperature maintained between 20 and 24 °C, and humidity between 20 and 60%).

**Table 4:** Surgical infection prevention information

Characteristic	Aqueous-PREP, ***N*** = 14*	PREPARE, ***N*** = 20
Number of infection preventionists per 250 hospital beds, median (IQR)	2.2 (1.7)	2.6 (1.9)
Infection preventionists certified in infection control, median (IQR)	3 (2.8)	4 (3)
Perform active surveillance cultures for MRSA in open fracture patients, *n* (%)	9 (64)	4 (20)
Perform active surveillance cultures for VRE in open fracture patients, *n* (%)	0 (0)	4 (20)
Perform active surveillance cultures for other organisms in patients with open fractures, *n* (%)	2 (14)	3 (15)
Perform active surveillance cultures for MRSA in closed fracture patients, *n* (%)	–	3 (15)
Perform active surveillance cultures for VRE in closed fracture patients, *n* (%)	–	3 (15)
Perform active surveillance cultures for other organisms in patients with closed fractures, *n* (%)	–	1 (5)
Decolonization protocol for MRSA, *n* (%)	9 (64)	12 (60)
Policies and/or guidelines on maintaining normothermia during perioperative period, *n* (%)	14 (100)	16 (80)
Airflow system(s) in operating room, *n* (%)**		
None	0 (0)	1 (5)
Vertical laminar flow	9 (64)	14 (70)
Conventional ventilation	6 (43)	7 (35)
Other	0 (0)	
Parameters in place in hospital operating rooms, *n* (%)**		
High-efficiency particulate air (HEPA) filters	12 (86)	15 (75)
Operating rooms are positive pressure	14 (100)	16 (80)
Air changes per hour	13 (93)	17 (85)
Temperature maintained between 20 and 24 °C and humidity between 20 and 60%	13 (93)	18 (90)

*The cluster that was withdrawn is not included

**Percentages do not sum to 100 as categories are not mutually exclusive

Orthopedic practices participating in the PREPARE trial employ a median of 2.6 infection preventionists per 250 hospital beds. Twenty percent of orthopedic practices (*n* = 4) perform active surveillance for MRSA in open fracture patients, and 15% of orthopedic practices (*n* = 3) perform active surveillance in closed fracture patients. No clusters perform active surveillance for VRE in either open or closed fracture patients. Sixty percent (*n* = 12) of orthopedic practices have a decolonization protocol for MRSA. Additionally, all practices had some infection control parameters in place in their operating rooms.

## Discussion

**Fig. 4:**
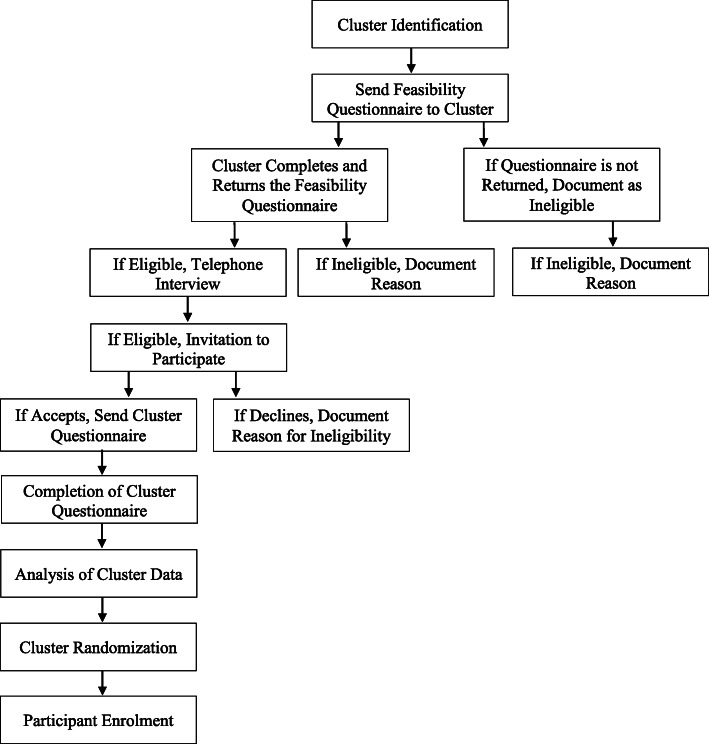
Overview of the PREP-IT cluster identification, selection, and randomization processes

The Aqueous-PREP and PREPARE trials represent two ongoing CRXO trials within the field of orthopedics. The successes we have experienced with cluster identification and selection provide support for the feasibility of using this trial design in orthopedic trauma trials. As part of the PREP-IT program’s commitment to transparency and upholding rigorous reporting standards, this paper clearly defines the cluster unit, provides detailed information about the cluster identification and selection process, and describes key characteristics of the included clusters (Fig. 4). These are some of the key reporting requirements described in the CONSORT extension for CRTs [4].

Given that the Aqueous-PREP and PREPARE trials use a pragmatic design, when selecting orthopedic practices (i.e., clusters), we aimed to include a sample that was representative of the orthopedic practices that treat the patient population in “real world” usual care settings. This is an essential component of pragmatic trials which aim to determine how well interventions work in the “real world” as opposed to explanatory trials which aim to determine how well interventions can work in ideal settings [8]. To accomplish this goal, we designed our cluster eligibility criteria to be as broad as possible while still ensuring that participating orthopedic practices would have the infrastructure and patient volume to successfully follow the protocol and meet study timelines. Through our cluster selection process, we identified 58 potentially eligible orthopedic practices and ultimately selected 5 sites to participate in both the Aqueous-PREP and PREPARE trials, 10 in the Aqueous-PREP trial only, and 15 in the PREPARE trial only. This represents an inclusion rate of 26% and 34%, respectively. Using the Pragmatic-Explanatory Continuum Indicator Summary (PRECIS-2) toolkit, a tool designed to measure where a trial falls on the pragmatic versus explanatory continuum on several domains, the setting of the Aqueous-PREP and PREPARE trials received a score of 4 out of a possible 5 indicating that the trials are “mostly pragmatic.” This score is reflective of the fact that most of our included orthopedic practices are affiliated with teaching hospitals that are level I trauma centers and regional referral centers versus a mix of trauma centers and local community hospitals. This is unsurprising as large academic hospitals tend to have both the infrastructure and patient volume to make participation in the PREP-IT trials feasible, whereas smaller community hospitals may have insufficient research infrastructure.

Despite our rigorous cluster selection process, we withdrew one cluster as they were unable to comply with the Aqueous-PREP protocol [6]. Specifically, this cluster was unable to meet the a priori threshold 90% of patients receiving the allocated antiseptic preparation solution. Additionally, the clinical site was unable to complete the case report forms, which led to incomplete data submission. These issues were the result of changes in research infrastructure from the time of site selection to site initiation. The Executive Committee, with support from the Methodology Core, made the decision to withdraw this site. As the criteria for cluster withdrawal were established prior to the initiation of the trial and a small proportion of data are affected, bias should not be introduced as a result of the decision to withdraw this cluster. Additionally, withdrawing this cluster will improve the overall integrity of the trial, as allowing them to continue in Aqueous-PREP would have led to increased protocol violations, treatment contaminations, and missing data.

As the primary outcome for both the Aqueous-PREP and PREPARE trials is SSI, we documented infection control processes. To remain pragmatic with data collection, we documented the infection control processes at three different levels: cluster level, surgeon level, and patient level. The most important SSI prevention strategies and infection strategies that are likely to vary from patient-to-patient, including antibiotic prophylaxis and blood sugar control, are documented at the patient level on the case report forms. Other infection control details (e.g., hair removal methods, type of surgical gown) are collected at the surgeon level, as these typically vary by surgeon but remain consistent across patients. Infection control measures that are implemented at the hospital level, including normothermia and decolonization protocols, are documented at the cluster level.

We aimed to select orthopedic practices that follow infection prevention protocols that are representative of the usual care that fracture patients would receive in the real world. We have found some variation in the infection control processes across the clusters in both the Aqueous-PREP trial and the PREPARE trial. Specifically, we found that 64% of all orthopedic practices in the Aqueous-PPREP trial perform active surveillance cultures for MRSA (64%). In the PREPARE trial, we found that only 20% of orthopedic practices perform active surveillance for MRSA in open fracture patients and 15% for closed fracture patients. In both trials, there was substantial variation in the use of decolonization protocols for MRSA (protocols in use at 64% of Aqueous-PREP practices and 60% of PREPARE practices).

Providing the knowledge user with a high level of detail regarding the hospital characteristics, orthopedic surgery details, and infection prevention protocols and procedures allows the knowledge user to determine the generalizability of the trial and the applicability to their hospital and surgical practice.

## Conclusions

When undertaking CRXO trials, it is imperative to follow rigorous reporting standards, as outlined in the CONSORT extensions for CRTs and CRXO trials, including providing knowledge users with a clear definition of the cluster unit, a thorough description of the cluster identification and selection process, and sufficient description of key cluster characteristics. The PREP-IT trials illustrate how this can be accomplished using two large infection prevention CRXO trials in orthopedic fracture patients as an example.

### Supplementary information

**Additional file 1.** THE PREP-IT Investigators.

## Data Availability

The datasets used and/or analyzed during the current study are available from the corresponding author on reasonable request.

## Appendix

### Characteristics of the withdrawn cluster

**Table 5:** Cluster characteristics

Characteristic	Withdrawn cluster
Level of trauma care provided	Level I
Ownership of hospital	Non-profit, not religious order affiliated
Hospital affiliation	Hospital system, free-standing
Population size served	6,000,000
Number of inpatient beds	640
Number of operating rooms	17
Number of beds in intensive care unit	102
Hospital is a primary teaching hospital for a medical school	Yes

**Table 6:** Surgical infection prevention information

Characteristic	Withdrawn cluster
Number of infection preventionists per 250 hospital beds	5
Infection preventionists certified in infection control	1
Perform active surveillance cultures for MRSA in open fracture patients	No
Perform active surveillance cultures for VRE in open fracture patients	No
Perform active surveillance cultures for other organisms in patients with open fractures	No
Decolonization protocol for MRSA	Yes
Policies and/or guidelines on maintaining normothermia during perioperative period	Yes
Airflow system(s) in operating room	Conventional ventilation
Parameters in place in hospital operating rooms	High-efficiency particulate air (HEPA) filters
	Operating rooms are positive pressure
	Air changes per hour
	Temperature maintained between 20 and 24 °C and humidity between 2 and 60%

## Abbreviations

PREP-IT Program: The Program of Randomized trials to Evaluate Pre-operative antiseptic skin solutions in orthopaedic Trauma
RCTs: Randomized controlled trials
CRXO: Cluster randomized crossover
SSI: Surgical site infection
Aqueous-PREP: A Pragmatic Randomized trial Evaluating Pre-operative aqueous antiseptic skin solutions in open fractures
PREPARE: A Pragmatic Randomized trial Evaluating Pre-operative Alcohol skin solutions in Fractured Extremities
CDC: Center for Disease Control and Prevention
IQR: Interquartile range
MRSA: Methicillin-resistant *Staphylococcus aureus*
VRE: Vancomycin-resistant *Enterococcus*
HEPA: High-efficiency particulate air
PRECIS-2: Pragmatic-Explanatory Continuum Indicator Summary
