# Correction to: Cluster identification, selection, and description in Cluster randomized crossover trials: the PREP-IT trials

**DOI:** 10.1186/s13063-020-04767-4

**Published:** 2020-09-30

**Authors:** Sheila Sprague, Taryn Scott, Shannon Dodds, David Pogorzelski, Paula McKay, Anthony D. Harris, Amber Wood, Lehana Thabane, Mohit Bhandari, Samir Mehta, Greg Gaski, Christina Boulton, Francesc Marcano-Fernández, Ernesto Guerra-Farfán, Joan Hebden, Lyndsay M. O’Hara, Gerard P. Slobogean, on behalf of the PREP-IT Investigators, Gerard P. Slobogean, Sheila Sprague, Jeffrey Wells, Mohit Bhandari, Jean-Claude D’Alleyrand, Anthony D. Harris, Daniel C. Mullins, Lehana Thabane, Amber Wood, Gregory J. Della Rocca, Joan Hebden, Kyle J. Jeray, Lucas Marchand, Lyndsay M. O’Hara, Robert Zura, Michael J. Gardner, Jenna Blasman, Jonah Davies, Stephen Liang, Monica Taljaard, P. J. Devereaux, Gordon H. Guyatt, Diane Heels-Ansdell, Debra Marvel, Jana Palmer, Jeff Friedrich, Nathan N. O’Hara, Frances Grissom, I. Leah Gitajn, Saam Morshed, Robert V. O’Toole, Bradley A. Petrisor, Megan Camara, Franca Mossuto, Manjari G. Joshi, Justin Fowler, Jessica Rivera, Max Talbot, Shannon Dodds, Alisha Garibaldi, Silvia Li, Uyen Nguyen, David Pogorzelski, Alejandra Rojas, Taryn Scott, Gina Del Fabbro, Olivia Paige Szasz, Paula McKay, Andrea Howe, Joshua Rudnicki, Haley Demyanovich, Kelly Little, C. Daniel Mullins, Michelle Medeiros, Eric Kettering, Diamond Hale, Andrew Eglseder, Aaron Johnson, Christopher Langhammer, Christopher Lebrun, Theodore Manson, Jason Nascone, Ebrahim Paryavi, Raymond Pensy, Andrew Pollak, Marcus Sciadini, Yasmin Degani, Haley K. Demyanovich, Katherine Joseph, Brad A. Petrisor, Herman Johal, Bill Ristevski, Dale Williams, Matthew Denkers, Krishan Rajaratnam, Jamal Al-Asiri, Jordan Leonard, Francesc A. Marcano-Fernández, Jodi Gallant, Federico Persico, Marko Gjorgjievski, Annie George, Roman M. Natoli, Greg E. Gaski, Todd O. McKinley, Walter W. Virkus, Anthony T. Sorkin, Jan P. Szatkowski, Joseph R. Baele, Brian H. Mullis, Lauren C. Hill, Andrea Hudgins, Patrick Osborn, Sarah Pierrie, Eric Martinez, Joseph Kimmel, John D. Adams, Michael L. Beckish, Christopher C. Bray, Timothy R. Brown, Andrew W. Cross, Timothy Dew, Gregory K. Faucher, Richard W. Gurich, David E. Lazarus, S. John Millon, M. Jason Palmer, Scott E. Porter, Thomas M. Schaller, Michael S. Sridhar, John L. Sanders, L. Edwin Rudisill, Michael J. Garitty, Andrew S. Poole, Michael L. Sims, Clark M. Walker, Robert M. Carlisle, Erin Adams Hofer, Brandon S. Huggins, Michael D. Hunter, William A. Marshall, Shea Bielby Ray, Cory D. Smith, Kyle M. Altman, Julia C. Bedard, Markus F. Loeffler, Erin R. Pichiotino, Austin A. Cole, Ethan J. Maltz, Wesley Parker, T. Bennett Ramsey, Alex Burnikel, Michael Colello, Russell Stewart, Jeremy Wise, M. Christian Moody, Stephanie L. Tanner, Rebecca G. Snider, Christine E. Townsend, Kayla H. Pham, Abigail Martin, Emily Robertson, Theodore Miclau, Utku Kandemir, Meir Marmor, Amir Matityahu, R. Trigg McClellan, Eric Meinberg, David Shearer, Paul Toogood, Anthony Ding, Erin Donohue, Tigist Belaye, Eleni Berhaneselase, Alexandra Paul, Kartik Garg, Joshua L. Gary, Stephen J. Warner, John W. Munz, Andrew M. Choo, Timothy S. Achor, Milton L. “ Chip” Routt, Mayank Rao, Guillermo Pechero, Adam Miller, Jennifer E. Hagen, Matthew Patrick, Richard Vlasak, Thomas Krupko, Kalia Sadasivan, Chris Koenig, Daniel Bailey, Daniel Wentworth, Chi Van, Justin Schwartz, Niloofar Dehghan, Clifford B. Jones, J. Tracy Watson, Michael McKee, Ammar Karim, Michael Talerico, Debra L. Sietsema, Alyse Williams, Tayler Dykes, William T. Obremskey, Amir Alex Jahangir, Manish Sethi, Robert Boyce, Daniel J. Stinner, Phillip Mitchell, Karen Trochez, Andres Rodriguez, Vamshi Gajari, Elsa Rodriguez, Charles Pritchett, Christina Boulton, Jason Lowe, Jason Wild, John T. Ruth, Michel Taylor, Andrea Seach, Sabina Saeed, Hunter Culbert, Alejandro Cruz, Thomas Knapp, Colin Hurkett, Maya Lowney, Michael Prayson, Indresh Venkatarayappa, Brandon Horne, Jennifer Jerele, Linda Clark, Francesc Marcano-Fernández, Montsant Jornet-Gibert, Laia Martínez-Carreres, David Martí-Garín, Jorge Serrano-Sanz, Joel Sánchez-Fernández, Matsuyama Sanz-Molero, Alejandro Carballo, Xavier Pelfort, Francesc Acerboni-Flores, Anna Alavedra-Massana, Neus Anglada-Torres, Alexandre Berenguer, Jaume Cámara-Cabrera, Ariadna Caparros-García, Ferran Fillat-Gomà, Ruben Fuentes-López, Ramona Garcia-Rodriguez, Nuria Gimeno-Calavia, Guillem Graells-Alonso, Marta Martínez-Álvarez, Patricia Martínez-Grau, Raúl Pellejero-García, Ona Ràfols-Perramon, Juan Manuel Peñalver, Mònica Salomó Domènech, Albert Soler-Cano, Aldo Velasco-Barrera, Christian Yela-Verdú, Mercedes Bueno-Ruiz, Estrella Sánchez-Palomino, Ernesto Guerra-Farfán, Yaiza García, Nicholas M. Romeo, Heather A. Vallier, Mary A. Breslin, Joanne Fraifogl, Eleanor S. Wilson, Leanne K. Wadenpfuhl, Paul G. Halliday, Darius G. Viskontas, Kelly L. Apostle, Dory S. Boyer, Farhad O. Moola, Bertrand H. Perey, Trevor B. Stone, H. Michael Lemke, Mauri Zomar, Ella Spicer, Chen “Brenda” Fan, Kyrsten Payne, Kevin Phelps, Michael Bosse, Madhav Karunakar, Laurence Kempton, Stephen Sims, Joseph Hsu, Rachel Seymour, Christine Churchill, Claire Bartel, Robert Miles Mayberry, Maggie Brownrigg, Cara Girardi, Ada Mayfield, Robert A. Hymes, Cary C. Schwartzbach, Jeff E. Schulman, A. Stephen Malekzadeh, Michael A. Holzman, Lolita Ramsey, James S. Ahn, Farhanaz Panjshiri, Sharmistha Das, Antoinisha D. English, Sharon M. Haaser, Jaslynn A. N. Cuff, Holly Pilson, Eben A. Carroll, Jason J. Halvorson, Sharon Babcock, J. Brett Goodman, Martha B. Holden, Debra Bullard, Wendy Williams, Thomas F. Higgins, Justin M. Haller, David L. Rothberg, Ashley Neese, Mark Russell, Marcus Coe, Kevin Dwyer, Devin S. Mullin, Clifford A. Reilly, Peter DePalo, Amy E. Hall, Marilyn Heng, Mitchel B. Harris, R. Malcolm Smith, David W. Lhowe, John G. Esposito, Mira Bansal, Patrick F. Bergin, George V. Russell, Matthew L. Graves, John Morellato, Heather K. Champion, Leslie N. Johnson, Sheketha L. McGee, Eldrin L. Bhanat, Samir Mehta, Derek Donegan, Jaimo Ahn, Annamarie Horan, Mary Dooley, Ashley Kuczinski, Ashley Iwu, David Potter, Robert VanDemark, Branden Pfaff, Troy Hollinsworth, Michael J. Weaver, Arvind G. von Keudell, Michael F. McTague, Elizabeth M. Allen, Todd Jaeblon, Robert Beer, Mark J. Gage, Rachel M. Reilly, Cindy Sparrow

**Affiliations:** 1 Department of Surgery, Division of Orthopaedic Surgery, McMaster University, 293 Wellington Street North, Suite 110, Hamilton, ON L8L 8E7 Canada; 2 Department of Health Research Methods, Evidence, and Impact, McMaster University, 293 Wellington St. N., Suite 110, Hamilton, ON L8L 8E7 Canada; 3 Department of Epidemiology and Public Health, University of Maryland School of Medicine, Baltimore, MD USA; 4 Association of periOperative Registered Nurses, Denver, CO USA; 5 Department of Orthopaedic Surgery, University of Pennsylvania Perelman School of Medicine, Philadelphia, PA USA; 6 Department of Orthopaedic Surgery, Indiana University School of Medicine, Indianapolis, IN USA; 7 Department of Orthopaedics, Banner Health and the University of Arizona-Tucson, Tucson, AZ USA; 8 Orthopedic Department, Parc Taulí Hospital Universitari, Institut d’Investigació i Innovació Parc Taulí I3PT, Universitat Autònoma de Barcelona, Sabadell, Spain; 9 Department of Orthopaedic Surgery and Traumatology, University Hospital Vall d’Hebron, Barcelona, Spain; 10 Department of Medicine, University of Maryland School of Medicine, Baltimore, MD USA; 11 Department of Orthopaedics, University of Maryland, R Adams Cowley Shock Trauma Center, Baltimore, MD USA

**Correction to: Trials 21, 712 (2020)**

**http://orcid.org/10.1186/s13063-020-04611-9**

Following publication of the original article [1], we were notified that the PREP-IT Investigators were not listed as contributors. They are all listed in Supplementary file 1 linked to this erratum.

## Supplementary information

**Additional file 1.**
