# Supplementary material for: Cluster identification, selection, and description in cluster randomized crossover trials: the PREP-IT trials
Source: Trials. 2020 Aug 12;21:712. doi: 10.1186/s13063-020-04611-9 (PMC7425374; doi:10.1186/s13063-020-04611-9)
Supplement: Supplementary file 1 — Additional file 1. THE PREP-IT Investigators. [file 13063_2020_4611_MOESM1_ESM.docx]

***THE PREP-IT Investigators**

**Executive Committee:** Gerard P. Slobogean (Principal Investigator, University of Maryland School of Medicine, Baltimore, MD); Sheila Sprague (Principal Investigator, McMaster University, Hamilton, ON); Jeffrey Wells (Patient Representative, Trauma Survivors Network, Falls Church, VA); Mohit Bhandari (Principal Investigator, McMaster University, Hamilton, ON)

**Steering Committee:** Gerard P. Slobogean (Co-Chair, University of Maryland School of Medicine, Baltimore, MD); Mohit Bhandari (Co-Chair, McMaster University, Hamilton, ON); Sheila Sprague (Principal Investigator, McMaster University, Hamilton, ON); Jean-Claude D’Alleyrand (Walter Reed National Military Medical Center, Bethesda, MD); Anthony D. Harris (University of Maryland School of Medicine, Baltimore, MD); Daniel C. Mullins (University of Maryland, Baltimore, MD); Lehana Thabane (McMaster University, Hamilton, ON); Jeffrey Wells (Trauma Survivors Network, Falls Church, VA); Amber Wood (Association of periOperative Registered Nurses, Denver, CO)

**Adjudication Committee:** Gregory J. Della Rocca (Chair, University of Missouri, Columbia, MO); Anthony D. Harris, (University of Maryland School of Medicine, Baltimore, MD); Joan Hebden (University of Maryland, Baltimore, MD); Kyle J. Jeray (Greenville Health System, Greenville, SC); Lucas Marchand (University of Maryland, Baltimore, MD); Lyndsay M. O’Hara (University of Maryland School of Medicine, Baltimore, MD); Robert Zura (LSU Health, New Orleans, LA)

**Data and Safety Monitoring Committee:** Michael J. Gardner (Chair, Stanford University School of Medicine, Palo Alto, CA); Jenna Blasman (Patient Representative, Kitchener, ON); Jonah Davies (University of Washington, Seattle, WA); Stephen Liang (Washington University, St. Louis, MO); Monica Taljaard (Ottawa Hospital Research Institute, Ottawa, ON)

**Research Methodology Core:** PJ Devereaux (McMaster University, Hamilton, ON); Gordon H. Guyatt (McMaster University, Hamilton, ON); Lehana Thabane (McMaster University, Hamilton, ON); Diane Heels-Ansdell (McMaster University, Hamilton, ON)

**Patient Centred Outcomes Core:** Debra Marvel (Patient Representative, Baltimore, MD); Jana Palmer (Patient Representative, Baltimore, MD); Jeffrey Wells (Patient, Trauma Survivors Network, Falls Church, VA); Jeff Friedrich (Editor, Slate Magazine, Washington DC); Daniel C. Mullins (University of Maryland, Baltimore, MD); Nathan N. O’Hara (University of Maryland School of Medicine, Baltimore, MD); Ms. Frances Grissom (Trauma Survivor Network, Baltimore, MD)

**Orthopaedic Surgery Core:** Gregory J. Della Rocca (University of Missouri, Columbia, MO); I. Leah Gitajn (Dartmouth University, Hanover, NH); Kyle J. Jeray (Greenville Health System, Greenville, SC); Saam Morshed (San Francisco General Hospital, San Francisco, CA); Robert V. O’Toole (University of Maryland School of Medicine, Baltimore, MD); Bradley A. Petrisor (Hamilton Health Science, Hamilton, ON)

**Operating Room Core:** Megan Camara (R Adams Cowley Shock Trauma Center, Baltimore, MD); Franca Mossuto (Hamilton Health Science, Hamilton, ON)

**Infectious Disease Core:** Anthony D. Harris (University of Maryland School of Medicine, Baltimore, MD); Manjari G. Joshi (University of Maryland School of Medicine, Baltimore, MD)

**Military Core:** Jean-Claude D’Alleyrand (Walter Reed National Military Medical Center, Bethesda, MD); Justin Fowler (United States Army, USA); Jessica Rivera (San Antonio Military Medical Center, San Antonio, TX); Max Talbot (Canadian Armed Forces, Montreal, QC)

**McMaster University Methods Center** (Hamilton, ON): Sheila Sprague (Principal Investigator); Mohit Bhandari (Principal Investigator); Shannon Dodds (Research Coordinator); Alisha Garibaldi (Research Coordinator); Silvia Li (Research Coordinator); Uyen Nguyen (Research Coordinator); David Pogorzelski (Research Coordinator); Alejandra Rojas (Research Coordinator); Taryn Scott (Research Coordinator); Gina Del Fabbro (Research Assistant); Olivia Paige Szasz (Research Assistant); Diane Heels-Ansdell (Statistician); Paula McKay (Manager)

**University of Maryland School of Medicine Administrative Center** (Baltimore, MD): Gerard P. Slobogean (Principal Investigator); Nathan N. O’Hara (Manager); Andrea Howe (Project Manager); Joshua Rudnicki (Project Manager); Haley Demyanovich (Project Manager); Kelly Little (Financial Manager)

**University of Maryland School of Pharmacy, The PATIENTS Program** (Baltimore, MD): C. Daniel Mullins (Executive Director); Michelle Medeiros (Director of Research); Eric Kettering (Senior Instructional Technology and Dissemination Specialist); Diamond Hale (Project Manager)

**PREP-IT Clinical Sites:**

*Lead Clinical Site (Aqueous-PREP and PREPARE):*

**University of Maryland School of Medicine, R Adams Cowley Shock Trauma Center, Baltimore, MD**: Robert V. O'Toole, Jean-Claude D'Alleyrand, Andrew Eglseder, Aaron Johnson, Christopher Langhammer, Christopher Lebrun, Theodore Manson, Jason Nascone, Ebrahim Paryavi, Raymond Pensy, Andrew Pollak, Marcus Sciadini, Gerard P. Slobogean, Yasmin Degani, Haley K. Demyanovich, Andrea Howe, Nathan N. O’Hara, Katherine Joseph, Joshua Rudnicki, Megan Camara

*Aqueous-PREP and PREPARE:*

**Hamilton Health Sciences – General Site, Hamilton, ON**: Brad A. Petrisor, Herman Johal, Bill Ristevski, Dale Williams, Matthew Denkers, Krishan Rajaratnam, Jamal Al-Asiri, Jordan Leonard, Francesc A. Marcano-Fernández*, Jodi Gallant, Federico Persico, Marko Gjorgjievski, Annie George

**IU Health Methodist Hospital, Indianapolis, IN:** Roman M. Natoli, Greg E. Gaski, Todd O. McKinley, Walter W. Virkus, Anthony T. Sorkin, Jan P. Szatkowski, Joseph R. Baele, Brian H. Mullis, Lauren C. Hill, Andrea Hudgins, Methodist OR Core II Staff

**San Antonio Military Medical Center, San Antonio, TX:** Patrick Osborn, Justin Fowler, Sarah Pierrie, Eric Martinez, Joseph Kimmel

**Prisma Health - Upstate, Greenville, SC**: Kyle J. Jeray, John D. Adams, Michael L. Beckish, Christopher C. Bray, Timothy R. Brown, Andrew W. Cross, Timothy Dew, Gregory K. Faucher, Richard W. Gurich Jr, David E. Lazarus, S. John Millon, M. Jason Palmer, Scott E. Porter, Thomas M. Schaller, Michael S. Sridhar, John L. Sanders, L. Edwin Rudisill, Jr, Michael J. Garitty, Andrew S. Poole, Michael L. Sims, Clark M. Walker, Robert M. Carlisle II, Erin Adams Hofer, Brandon S. Huggins, Michael D. Hunter, William A. Marshall, Shea Bielby Ray, Cory D. Smith, Kyle M. Altman, Julia C. Bedard, Markus F. Loeffler, Erin R. Pichiotino, Austin A. Cole, Ethan J Maltz, Wesley Parker, T. Bennett Ramsey, Alex Burnikel, Michael Colello, Russell Stewart, Jeremy Wise, M. Christian Moody, Stephanie L. Tanner, Rebecca G. Snider, Christine E. Townsend, Kayla H. Pham, Abigail Martin, Emily Robertson

**University of California, San Francisco, San Francisco, CA:** Saam Morshed, Theodore Miclau, Utku Kandemir, Meir Marmor, Amir Matityahu, R. Trigg McClellan, Eric Meinberg, David Shearer, Paul Toogood, Anthony Ding, Erin Donohue, Tigist Belaye, Eleni Berhaneselase, Alexandra Paul*, Kartik Garg

*Aqueous-PREP:*

**McGovern Medical School at UTHealth Houston, Houston, TX**: Joshua L. Gary, Stephen J Warner, John W. Munz, Andrew M. Choo, Timothy S. Achor, Milton L. “Chip” Routt, Mayank Rao, Guillermo Pechero, Adam Miller*

**University of Florida, Gainesville, FL:** Jennifer E. Hagen, Matthew Patrick, Richard Vlasak, Thomas Krupko, Kalia Sadasivan*, Chris Koenig, Daniel Bailey*, Daniel Wentworth*, Chi Van, Justin Schwartz

**The CORE Institute, Phoenix, AZ**: Niloofar Dehghan, Clifford B Jones*, J Tracy Watson, Michael McKee, Ammar Karim*, Michael Talerico, Debra L Sietsema, Alyse Williams, Tayler Dykes

**Vanderbilt Medical Center, Nashville, TN**: William T Obremskey, Amir Alex Jahangir, Manish Sethi, Robert Boyce, Daniel J. Stinner, Phillip Mitchell, Karen Trochez, Andres Rodriguez*, Vamshi Gajari, Elsa Rodriguez, Charles Pritchett

**Banner University Medical Center – Tucson, Tucson, AZ:** Christina Boulton, Jason Lowe, Jason Wild*, John T. Ruth, Michel Taylor, Andrea Seach, Sabina Saeed, Hunter Culbert, Alejandro Cruz, Thomas Knapp*, Colin Hurkett*, Maya Lowney

**Wright State University, Dayton, OH**: Michael Prayson, Indresh Venkatarayappa, Brandon Horne, Jennifer Jerele, Linda Clark

**Hospital Universitari Parc Tauli, Barcelona, Spain:** Francesc Marcano-Fernández, Montsant Jornet-Gibert, Laia Martínez-Carreres, David Martí-Garín, Jorge Serrano-Sanz,

Joel Sánchez-Fernández, Matsuyama Sanz-Molero, Alejandro Carballo, Xavier Pelfort, Francesc Acerboni-Flores, Anna Alavedra-Massana, Neus Anglada-Torres, Alexandre Berenguer, Jaume Cámara-Cabrera, Ariadna Caparros-García, Ferran Fillat-Gomà, Ruben Fuentes-López, Ramona Garcia-Rodriguez, Nuria Gimeno-Calavia, Guillem Graells-Alonso, Marta Martínez-Álvarez, Patricia Martínez-Grau, Raúl Pellejero-García, Ona Ràfols-Perramon, Juan Manuel Peñalver, Mònica Salomó Domènech, Albert Soler-Cano, Aldo Velasco-Barrera, Christian Yela-Verdú, Mercedes Bueno-Ruiz, Estrella Sánchez-Palomino

**Vall d’Hebron Hospital, Barcelona, Spain:** Ernesto Guerra-Farfán, Yaiza García

*PREPARE:*

**MetroHealth Medical Center, Cleveland, OH**: Nicholas M. Romeo, Heather A Vallier, Mary A Breslin*, Joanne Fraifogl, Eleanor S Wilson*, Leanne K Wadenpfuhl*, Paul G. Halliday

**FRASER HEALTH AUTHORITY/Royal Columbian Hospital, New Westminster, BC:** Darius G. Viskontas, Kelly L. Apostle, Dory S. Boyer, Farhad O. Moola, Bertrand H. Perey, Trevor B. Stone, H. Michael Lemke, Mauri Zomar, Ella Spicer, Chen “Brenda” Fan, Kyrsten Payne

**Carolinas Medical Center, Atrium Health Musculoskeletal Institute, Charlotte, NC**: Kevin Phelps, Michael Bosse, Madhav Karunakar, Laurence Kempton, Stephen Sims, Joseph Hsu, Rachel Seymour, Christine Churchill, Claire Bartel, Robert Miles Mayberry, Maggie Brownrigg, Cara Girardi, Ada Mayfield

**Inova Fairfax Medical Campus, Falls Church, VA:** Robert A. Hymes, Cary C. Schwartzbach, Jeff E. Schulman, A. Stephen Malekzadeh, Michael A. Holzman, Lolita Ramsey, James S. Ahn, Farhanaz Panjshiri*, Sharmistha Das, Antoinisha D. English, Sharon M. Haaser, Jaslynn A. N. Cuff

**Wake Forest Baptist Health, Winston-Salem, NC:** Holly Pilson, Eben A. Carroll, Jason J. Halvorson, Sharon Babcock, J. Brett Goodman, Martha B. Holden, Debra Bullard, Wendy Williams

**University of Utah, Salt Lake City, Utah**: Thomas F. Higgins, Justin M. Haller, David L. Rothberg, Ashley Neese, Mark Russell

**Dartmouth-Hitchcock Medical Center, Lebanon, NH:**I. Leah Gitajn, Marcus Coe, Kevin Dwyer, Devin S. Mullin, Clifford A. Reilly, Peter DePalo, Amy E. Hall

**Massachusetts General Hospital, Boston, MA**: Marilyn Heng, Mitchel B. Harris, R. Malcolm Smith, David W. Lhowe, John G. Esposito, Mira Bansal

**University of Mississippi Medical Center, Jackson, MS:** Patrick F. Bergin, George V. Russell, Matthew L. Graves, John Morellato, Heather K. Champion, Leslie N. Johnson, Sheketha L. McGee, Eldrin L. Bhanat

**University of Pennsylvania, Philadelphia, PA**: Samir Mehta, Derek Donegan, Jaimo Ahn, Annamarie Horan, Mary Dooley, , Ashley Kuczinski, Ashley Iwu

**Sanford Health, Sioux Falls, SD**: David Potter, Robert VanDemark III, Branden Pfaff, Troy Hollinsworth

**Brigham Women's Hospital, Boston, MA**: Michael J. Weaver, Arvind G. von Keudell, Michael F. McTague, Elizabeth M. Allen

**University of Maryland Prince George’s Capital Region Health: Cheverly MD**: Todd Jaeblon, Robert Beer, Haley K. Demyanovich

**Duke University Hospital, Durham, NC:** Mark J. Gage, Rachel M. Reilly, Cindy Sparrow

** Individual is no longer actively working on the Aqueous-PREP and / or PREPARE trial*
